# Chromosome Evolution in the Family Solanaceae

**DOI:** 10.3389/fpls.2021.787590

**Published:** 2022-01-28

**Authors:** Rocío Deanna, María Cristina Acosta, Marisel Scaldaferro, Franco Chiarini

**Affiliations:** ^1^Instituto Multidisciplinario de Biología Vegetal (CONICET-UNC), Córdoba, Argentina; ^2^Department of Ecology and Evolutionary Biology, University of Colorado at Boulder, Boulder, CO, United States

**Keywords:** comparative methods, cytogenetics, karyotype, nightshades, phylogeny

## Abstract

This review summarizes and discusses the knowledge of cytogenetics in Solanaceae, the tomato family, its current applications, and prospects for making progress in fundamental systematic botany and plant evolution. We compile information on basic chromosome features (number, size, morphology) and molecular cytogenetics (chromosome banding and rDNA patterns). These data were mapped onto the Solanaceae family tree to better visualize the changes in chromosome features and evaluate them in a phylogenetic context. We conclude that chromosomal features are important in understanding the evolution of the family, especially in delimiting clades, and therefore it is necessary to continue producing this type of data. The potential for future applications in plant biology is outlined. Finally, we provide insights into understanding the mechanisms underlying Solanaceae’s diversification that could substantially contribute to developing new approaches for future research.

## Introduction

Chromosomes provide information for inferring phylogenetic relationships, since they are hereditary elements of the whole nuclear genome and discrete hereditary units of mutation. Within taxa, they may vary in number, size, morphology, and staining properties (Weiss-Schneeweiss and Schneeweiss, 2013). Chromosome number has always been a common character employed, since it is the most easily obtained information and the only one that is known for most plant groups. Karyotypes represent an important aspect in plant speciation since chromosomal differences establish immediate postzygotic crossing barriers. They provide diagnostic characters for plant systematics and evolution that are usually expected to be congruent with clade divergence (e.g., Baltisberger and Hörandl, 2016). In Solanaceae, chromosomes have played a relevant role: they have been useful to delimit taxa (Camadro et al., 2012), for genetic studies with model species (Blakeslee et al., 1920; Avery et al., 1959), and for the selection of cultivars in economically important species (Pringle and Murray, 1992). With the advent of molecular phylogenetic techniques, chromosome data acquired a new value, to the point that within the family, there is a major lineage named as the “*x* = 12 clade,” whose members share such cytological synapomorphy (Olmstead et al., 2008). This review summarizes and discusses the knowledge of cytogenetics in Solanaceae, its current applications and prospects for making progress in fundamental systematic botany and plant evolution. Available chromosome data were reassessed by employing them in ancestral state reconstruction and thus revealing how many times traits changed over evolutionary time, inferring the degree of convergence, and suggesting relationships between chromosomes and other Solanaceae characteristics. Continuous cytogenetic characters (C, mean chromosome length; r, mean arm ratio or r index; TL, total haploid chromosome length; and m_ratio, the ratio between the number of metacentric to the total number of chromosomes) were mapped and plotted onto the family phylogeny of Särkinen et al. (2013) with modifications by Dupin et al. (2017), after removing those species without data using the drop.tip function of the package {ape} in R (Paradis et al., 2004). The ancestral states were estimated assuming that the species evolved under a Brownian model and the mapping was performed using the ContMap function of the package {phytools} in R version 3.4.2 (Revell, 2012; Supplementary Figures 1–4). All the methodologies to gather the data are in Supplementary Table 2.

## Chromosome Counts

Knowing the basic number *x* for all Angiosperms and its clades has been a recurring question (Darlington, 1956). Initially, it was considered of little taxonomic utility at high hierarchical levels (orders, families) due to homoplasy. For Solanales, Raven (1975) proposed *x* = 7 while *x* = 12 may have derived from the tetraploid level (*n* = 14) by aneuploid reduction in the early history of Solanaceae. Acosta et al. (2006) suggested *x* = 11 as the ancestral number that best fits the available phylogenetic hypotheses (Olmstead et al., 1999). Recently, models of chromosome evolution were customized using statistical frameworks, allowing to leverage other tools, like Ancestral State Reconstruction (ASR, FitzJohn, 2012), and to infer character evolution across phylogenetic trees (Revell, 2012). The compilation and analysis of data in a phylogenetic context allow us to recalculate the first estimates and reconstruct the history of the changes. Although the basic number x is homoplastic in Solanaceae, it is more conserved than other karyotype features, which makes it useful to delimit taxa at lower hierarchical levels: tribes, subtribes, genera (e.g., Rodríguez et al., 2020). The availability of chromosome data is essential to delve into these analyses, but the information is still scattered and fragmentary (Supplementary Tables 1, 2). Among the five largest genera of Solanaceae, the best studied (*Solanum*) has 48% of its species counted. There is a bias toward groups of species with economically valuable representatives (e.g., *Capsicum*, *Nicotiana*), while small clades and monotypic genera are karyologically unknown (Supplementary Tables 1, 2). Applying model approaches to chromosome number would clarify this topic (Glick and Mayrose, 2014), although such methodologies have difficulties given the size of the matrix for the entire family (Supplementary Table 1) and discussing such downsides is beyond the scope of this work.

Solanaceae have undergone whole genome duplications occurring near the time of its origin, which could have contributed to the rise of key traits and drove species diversification (Schranz et al., 2012). Polyploidy, a phenomenon constantly reviewed (e.g., Van de Peer et al., 2021), because of the advantages it may confer, has been a subject since the beginning of cytogenetics in Solanaceae (e.g., Blakeslee et al., 1920) up to the present (e.g., Hijmans et al., 2007; Chiarini et al., 2018). Nowadays, the availability of data makes it possible to analyze polyploidy throughout the evolutionary history of the family and relate it to colonization of new habitats, speciation events and to the rising of adaptive traits (Hijmans et al., 2007; Scaldaferro et al., 2012; Zenil-Ferguson et al., 2018, 2019; McCarthy et al., 2019).

## Karyotypes

Blakeslee’s pioneering work on *Datura* mutants (Blakeslee et al., 1920, and subsequent papers) allowed to identify pairs of homologs by their shape, and correlate alterations in meiotic behavior to exomorphology. The chromosome morphology observable with classical technique is still essential to detect rearrangements involved in speciation, for example, detection of allopolyploids in *Nicotiana* (Goodspeed, 1954), Robertsonian translocations in *Solanum* (Chiarini and Bernardello, 2006) and in *Chamaesaracha* (Rodríguez et al., 2020), and centric fission in *Capsicum* (Jarret et al., 2019). In contrast, uniform chromosome morphology may be the explanation for crossability among Iochrominae species (Deanna et al., 2018) and suggests the phenomenon of “karyotype orthoselection” in *Lycieae* (Bernardello et al., 2008). Karyotype data integrated with molecular phylogenies provide characters for Solanaceae systematics and evolution (e.g., *Nierembergia*: Acosta et al., 2016; *Jaborosa*, Chiarini et al., 2017; *Iochroma*, Deanna et al., 2018; *Physalis*, Rodríguez et al., 2020). However, the distribution of cytological studies is uneven among the genera of the family (Supplementary Table 2). We registered 420 species of Solanaceae with some kind of data (karyotype formulae, idiograms, asymmetry indices), which makes up a percentage of ca. 15% of the family. The most frequently informed characters are the following.

### Chromosome Size

**FIGURE 1:**
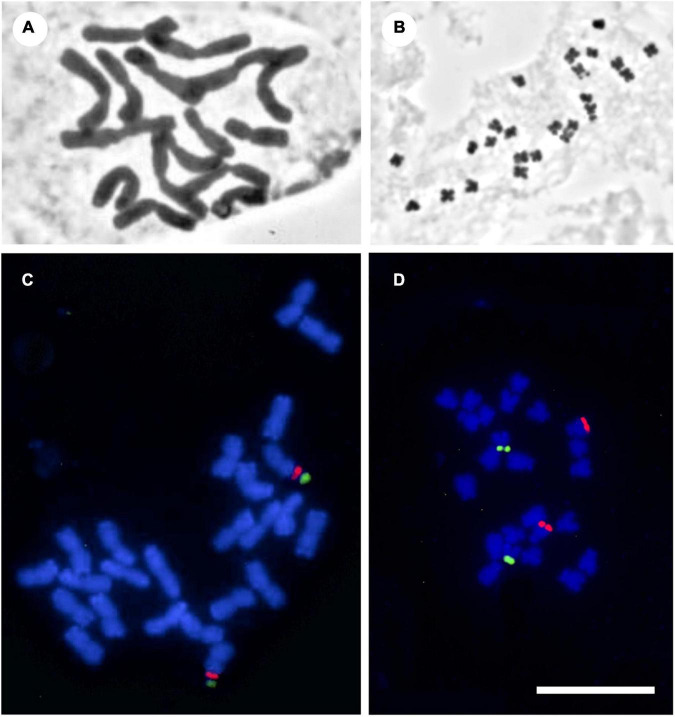
Photomicrographs of mitotic metaphases of Solanaceae, illustrating the chromosome variety found in the family. **(A)** *Cestrum buxifolium* Kunth (2n = 16, *c* = 11.5 μm, *r* = 1.4). **(B)** *Solanum lidii* Sunding (2n = 24, *c* = 1.3 μm, *r* = 1.6). **(C)** *Iochroma edule* S. Leiva (2n = 24, *c* = 4.0, *r* = 1.2). **(D)** *Physalis lagascae* Roem. & Schult. (2n = 24, *c* = 2.5 μm, *r* = 2.8). Panels **(A,B)** with classical staining, panels **(C,D)** fluorescence *in situ* hybridization with 5S (red) and 18-5.8-26S (green) rDNA probes. All at the same scale. Bar = 10 μm.

Chromosome size (usually expressed as length in μm) is useful to single out individuals, samples, populations or species. DNA content is directly related to it, so the alleged factors affecting the former may indirectly affect the latter as the speed of DNA replication and the duration of the life cycle. Also, long chromosomes would undergo a higher number of chiasmata than small ones (Turney et al., 2004) and low recombination due to small chromosomes would be compensated by high ploidy levels (Nakazato et al., 2006). In Solanaceae, average chromosome size varies from ca. 1 μm (in *Solanum* and *Atropa*) to 6.5–11.5 μm (in *Cestrum* and the Cyphomandra clade of *Solanum*, Figure 1, Supplementary Figure 1, and Supplementary Table 3) although most species have small to medium-sized chromosomes, with an overall mean chromosome length of 2.95 ± 1.78 (Supplementary Table 3). In order to better visualize the changes in chromosome size and evaluate them in a phylogenetic context, we mapped C and TL onto the Solanaceae family tree (Supplementary Tables 3, 4). Although these two variables are homoplastic (Supplementary Figures 1, 2) and have no significant phylogenetic signal, it is still useful to characterize some clades which are equivalent to medium-hierarchy taxonomic levels. Cestreae, Cyphomandra and *Capsicum* clades are outstanding because they have high C, conspicuously different from the remaining clades (Supplementary Figure 1). These synapomorphic large chromosomes arose three times separately and are an interesting case to study phenomena such as “genome obesity” (Bennetzen and Kellogg, 1997), increases in DNA content through the incorporation of heterochromatin, repetitive sequences, and retrotransposons (Fregonezi et al., 2007; Miguel et al., 2012; Scaldaferro et al., 2013).

### Chromosome Morphology

#### Asymmetry

There are a variety of indices to estimate chromosomal asymmetry and their accuracy is arguable (Peruzzi and Eroğlu, 2013). Here we have considered the r index because it is the most widespread, intuitive and easy to calculate, and also the most frequently informed (Supplementary Table 5). Asymmetry indices try to provide an idea of the general morphology of the karyotype through a single number, but logically not regarding the morphology of each chromosome. When all pairs of chromosomes undergo similar changes at the same time (like in *Nicotiana*, Kovarik et al., 2004), other measurements are needed to distinguish individual chromosome rearrangements. In a general survey of Solanaceae, Badr et al. (1997) reported values of r from 1.17 to 2.78, whereas in *Solanum* it ranges from 1.19 to 3.71 with an overall mean of 1.64 (Chiarini et al., 2018). Thus, karyotype asymmetry is not uniform in the family. According to ASR, symmetrical karyotypes are plesiomorphic (Supplementary Figure 3), being asymmetry a synapomorphy of some clades (*Physalis*, *Nicotiana*), while others would have conserved symmetry (*Lycium*). This supports the idea that in Solanaceae, shifts in chromosome morphology are frequent when considering broad frames of time (Wu and Tanksley, 2010; Chiarini et al., 2018).

#### Karyotype Formulae

The karyotype formula is a mean of expressing the result of a measurement process of the chromosome complement, i.e., the whole set of chromosomes in a nucleus, and it gives an overall idea of its morphology. Morphology provides the first clue to single out chromosome pairs (e.g., heteromorphic sex chromosomes). Chromosome morphology can be related to the plant mating system: in *Rumex*, a species with sex chromosomes, the suppression of recombination varies between metacentric and acrocentric chromosomes (e.g., Rifkin et al., 2021). Also, bimodal karyotypes visible with classical technique evidence the presence of two genomes (Brandham and Doherty, 1998). According to the widespread Levan’s classification (Levan et al., 1964), chromosomes can be, in increasing order of asymmetry, ***m***, ***sm***, ***st***, and ***t***. On this basis, karyotypes of Solanaceae are symmetrical, since only 17 out of the total species (4%) with known karyotypes contain 1–3 ***t*** chromosomes, while 100 (24%) present 1–7 ***sm*** chromosomes. Most Solanaceae have more than half of chromosomes per complement in the ***m*** and/or ***sm*** categories. These features are useful to characterize entire clades within the family: there are groups with a majority of ***m*** chromosomes, e.g., *Lycium* (Stiefkens et al., 2020), but there are also groups in which complements include ***st*** and ***t*** chromosomes, e.g., *Nicotiana*, *Capsicum*, *Jaborosa*, *Physalis* (Figure 1D), *Hyoscyamus*, *Nierembergia* (Sheidai et al., 1999; Moscone et al., 2006; Scaldaferro et al., 2013; Acosta et al., 2016; Chiarini et al., 2017; Rodríguez et al., 2020) and in some clades of *Solanum* (Bernardello et al., 1994; Acosta et al., 2005). Some *Nicotiana* species have karyotypes mostly with ***st*** chromosomes (Villa, 1984) and *Leptoglossis linifolia* has mostly ***sm*** chromosomes (Acosta et al., 2016).

**FIGURE 2:**
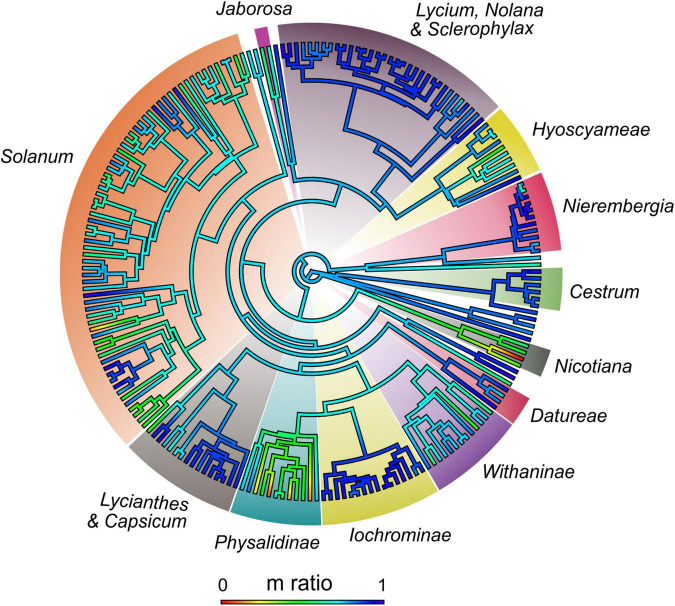
Heatmap of m ratio (proportion of metacentric chromosomes over the total chromosomes of each karyotype formula) reconstructed on Solanaceae. Scale below indicates values of the ratio and its color guide. Values vary from 0 (no metacentric chromosomes) to 1 (all chromosomes metacentric).

In Solanaceae, establishing the direction of karyotype evolution is unlikely, as many reversals might have occurred (Stace, 2000), and karyotype asymmetry might be a temporary state rather than an evolutionary end (Mandáková and Lysak, 2008). Comparing karyotype formulae is arguable because it is difficult to establish homologies of chromosome pairs among different species. Here, we calculated the proportion of ***m*** chromosomes for each nightshade with the available karyotype formula and mapped this ratio onto the phylogeny (Figure 2, Supplementary Figure 4, and Supplementary Table 6). This reconstruction suggests the most probable ancestral karyotypes had ± 80% of ***m*** chromosomes (i.e., symmetric karyotype). Formulae with few or no ***m*** chromosomes are autapomorphic (e.g., *Nicotiana plumbaginifolia*) while complete formulae with ***m*** chromosomes is a synapomorphy for certain clades (*Lycium*, Iochrominae, Figure 1C). Transitions between karyotype formulae can be interpreted as evidence of chromosome rearrangements. Particularly, formulae with ***st*** or ***t*** chromosomes are seemingly the result of a deletion or translocation of the entire or part of one arm (Weiss-Schneeweiss and Schneeweiss, 2013) or centric fissions (Moscone et al., 2006; Jarret et al., 2019).

### Chromosome Banding

Banding techniques are the next step in identifying chromosome pairs in a complement. They leverage on the physical properties of chromatin, using dyes that differentially stain euchromatin and fractions within heterochromatin (e.g., AT- or GC-rich), which are then visualized as bands across the chromatids. In Solanaceae, 218 spp. out of approx. 2,800 (ca. 7.7%) have been studied with any banding technique. The largest number of species studied belong to *Solanum* (69 out of 1,238 species, 5.6%), followed by *Capsicum* (25 out of 41, 60.9%) and *Lycium* (24 out of 105, 22.9%) (Supplementary Table 2). These last two genera are also the best studied in relative terms, together with *Nierembergia* (71.4% of 21 spp.), *Jaborosa* (65.2% of 23 spp.) and *Iochroma* (22.6% of 31 spp.) (Supplementary Table 2). A variety of banding techniques have been essayed in Solanaceae to answer a range of questions: silver impregnation detected active nucleolar organizing regions (NORs) (e.g., in *Cestrum*, Berg and Greilhuber, 1993; *Capsicum*, Scaldaferro et al., 2016; *Solanum*, Miguel et al., 2012; *Deprea*, Deanna et al., 2014); Giemsa C-banding revealed differences between taxa and contributed to taxonomic grouping of *Capsicum* (Moscone et al., 1993); Fluorescent banding, mostly with the double staining CMA/DAPI technique (Schweizer, 1976) demonstrated that chromosomes are mostly composed of non-coding chromatin, and also revealed strong phylogenetic signal, defining specific patterns in different clades (Acosta et al., 2016). CMA/DAPI technique helped to detect variability despite the morphological uniformity of the chromosomes, provided information on genetic variation at a population level regarding speciation, and revealed AT- and GC-rich regions of B chromosomes. Together with FISH (see below), this technique showed that B chromosomes possess nucleolar activity and nucleolar competition with the A chromosomes (Acosta and Moscone, 2011; Acosta et al., 2016; Montechiari et al., 2020).

Heterochromatin amount (H.a.) is a value often reported (usually as a percentage). It exhibits a remarkable variation among species of the same genus, for example in *Solanum* it varies from 1.86 to 35.43% (Brasileiro-Vidal et al., 2009; Chiarini et al., 2018). The data on H.a. have served to discuss whether the increases in genome size (either measured visually as TL, or as DNA content through flow cytometry) are due to increases in the amount of one or another fraction of chromatin (e.g., Karsburg et al., 2009). In some clades, H.a. is positively correlated with karyotype length (Moscone et al., 2006; Scaldaferro et al., 2013), while in others there is negative or no correlation, which is unexpected because heterochromatin is considered as an additional component of the genome (Pringle and Murray, 1993; Acosta et al., 2012, 2016). Heterochromatin patterns are more variable in some genera (*Jaborosa, Solanum*) than in other members of the *x* = 12 clade (e.g., *Lycium* and *Sclerophylax*), where heterochromatin is scarce and restricted to the NORs (Lujea and Chiarini, 2017; Stiefkens et al., 2020). Variable patterns might be evidence of intense chromosomal rearrangements (Evtushenko et al., 2016) associated with diversification and colonization of new habitats, since they function as species barriers (Hughes and Hawley, 2009).

### rDNA and Repetitive DNA Patterns

Ribosomal DNAs are fundamental components of all cell types. In most plants, 5S and 18S-5.8S-26S rDNAs are present in high copy numbers to satisfy the cellular requirement for ribosomes. These rDNAs, highly conserved in plants, are the commonest markers for FISH (Heslop-Harrison and Schwarzacher, 2011). They are repetitive, tandemly arranged and generally clustered at different loci, either with a spatial separation or also linked in a single unit. FISH enables direct visualization of rDNA loci on the chromosomes and determination of their rearrangements and organization (e.g., Srebniak et al., 2002; Melo and Guerra, 2003; Lou et al., 2010). Thus, homologous chromosomes in a complement can be identified and related species can be compared, answering evolutionary questions. Number, position and organization of rDNAs have been suitably employed in Solanaceae as systematic and evolutionary approaches (Figures 1C,D and Supplementary Table 2), for example in *Nierembergia* (Acosta et al., 2016), *Jaborosa* (Chiarini et al., 2017) and *Iochroma* (Deanna et al., 2018). Genera with economically important species (*Capsicum*, *Nicotiana*) have received special attention (Kitamura et al., 2005; Scaldaferro et al., 2016). Within the largest genera, *Lycium* is the most studied (Stiefkens et al., 2020), revealing uniformity of patterns, but little is known about the rest of the family. Chromosomal rearrangements between *Solanum* crops and several related species have been studied using tomato and potato bacterial artificial chromosomes (BACs) in multiple FISH essays, providing support for grouping of species into sections and suggesting interspecific hybridization events (Doganlar et al., 2002; Frary et al., 2016). Another type of repetitive DNA revealed by FISH are the telomeric sequences. Most plants present the Arabidopsis-type telomeres, but some Solanaceae are exceptional in lacking such sequences: the characterization of unusual telomeres in the three genera of Cestreae has shed light on patterns of telomere evolution, maintenance and function (Sýkorová et al., 2003; Peška et al., 2015).

## Cytogenetic and Phylogenetic Gaps

There is a growing interdependence between chromosomal data and molecular phylogeny. Cytogenetics and molecular phylogeny feed into each other, providing evidence in both ways. To some extent, Solanaceae ASRs allow reconstructing the cytogenetic data when there is molecular data (e.g., Rodríguez et al., 2020), but the reverse is obviously not possible. Thus, the largest information gaps are found in *Cestrum*, (165 species without any data of a total of 233, neither counts nor sequences), *Lycianthes* (116 species without data of about 161), *Jaltomata* (32 species of 73), *Physalis* (47 of 109) and *Browallia* (18 species without data of about 22). Of the ca. 2,900 species in the family, 494 (17%) possess chromosome counts and are included in the phylogeny of Särkinen et al. (2013), while of these, only 207 (7% of the family) also possess karyotypes. At the same time, there are 137 species for which there are counts but no sequences, and of these, 40 also have karyotypes, but these data cannot be analyzed in a molecular phylogenetic context (Supplementary Tables 7, 8). Important gaps in both cytogenetics and phylogeny still wait to be filled: in Goetzeoideae, a clade of which nothing is known; in Solandreae, only chromosome studies in one *Trianaea* species and two *Dyssochroma*; *Cuatresia*, genus of Physalideae with an important number of species about which nothing is known (Supplementary Table 8).

## Cytogenetics and Genomics in Solanaceae

Extensive pairwise comparative mapping studies have been performed for several major solanaceous crops relative to tomato (Tanksley et al., 1988, 1992; Livingstone et al., 1999; Doganlar et al., 2002; Wu et al., 2009; Wu and Tanksley, 2010). By using single-copy conserved ortholog sets (COSII), the outcomes of chromosomal evolution in the Solanaceae over 30 million years were deduced, estimating the rates and timing of chromosomal rearrangements as well as calculating the age of ancestral species and predicting their genome features (Wu and Tanksley, 2010). Thus, studies integrating cytogenetics and genomics, though focused on main crops and close relatives, generate knowledge that can be extrapolated to the rest of the species of the family.

Recently, Cytogenetics developed feedback with Genomics: the availability of species with complete chloroplast genome sequenced and some with complete nuclear genome, allows the finding of new markers for hybridization techniques. In return, these cytogenetic data become valuable information on chromosome identification and genome organization, enabling the spatial location of specific, single, or low-copy sequences. Next-generation sequencing speeds up cytogenetic research on Solanaceae. In species with available transcriptomes, like *S. dulcamara*, new markers like SNP SSR, AFLP and CAPS were developed, allowing the construction of a genome-wide genetic linkage map (D’Agostino et al., 2013). Based on gene orthologs, the markers were anchored to the genome of related *Solanum* species (tomato, potato and eggplant), revealing both conserved and novel chromosomal rearrangements. Estimates of the evolutionary moment of rearrangements were possible and showed that chromosomal breakpoints are regularly re-used.

Also, advances in NGS, such as the study of repetitive DNA, contributed to the design of more specific oligo-probes for FISH (Buggs et al., 2012), and hence bringing new chances to improve our understanding of systematics and genome organization at different taxonomic hierarchies. More recently, there has been progress in the phylogenomics of Solanaceae, particularly in some clades within the family, through the use of nuclear target capture and high-throughput sequencing of transcriptomic data (Gates et al., 2018; Poczai et al., 2018; Gagnon et al., 2021). These approaches provide more comprehensive and better resolved phylogenies which can be used in the study of chromosome evolution.

## Concluding Remarks

• Evolutionary trends: The ancestor of the family had a karyotype formula with 80% of m chromosomes, each about 2 μm in size. Increased asymmetry and size are synapomorphies of some clades that arose in independent events.
• Although homoplastic at a higher hierarchical level, karyotype traits are useful to delimit taxa at lower levels: tribes, subtribes, genera.
• Important gaps in both cytogenetics and phylogeny still wait to be filled.
• Potential for future applications: once there is a genomic phylogenetic backbone of Solanaceae, including divergence time estimates, adding chromosomes to it would be illuminating, because relationships between major clades of the family and Solanoideae are still poorly resolved using Sanger sequence data alone.

## Author Contributions

FC and RD conceived the ideas, designed the methodology, analyzed the data, and wrote the manuscript. FC, RD, MS, and MA collected the data. All authors contributed to the article and approved the submitted version.

## Conflict of Interest

The authors declare that the research was conducted in the absence of any commercial or financial relationships that could be construed as a potential conflict of interest.

## Publisher’s Note

All claims expressed in this article are solely those of the authors and do not necessarily represent those of their affiliated organizations, or those of the publisher, the editors and the reviewers. Any product that may be evaluated in this article, or claim that may be made by its manufacturer, is not guaranteed or endorsed by the publisher.
