# Supplementary material for: Chromosome Evolution in the Family Solanaceae
Source: Front Plant Sci. 2022 Jan 28;12:787590. doi: 10.3389/fpls.2021.787590 (PMC8832121; doi:10.3389/fpls.2021.787590)

Figure S2. Heatmap of Total Haploid Chromosome Length (TL) reconstructed on Solanaceae. Scale below indicates values of TL and its color guide.

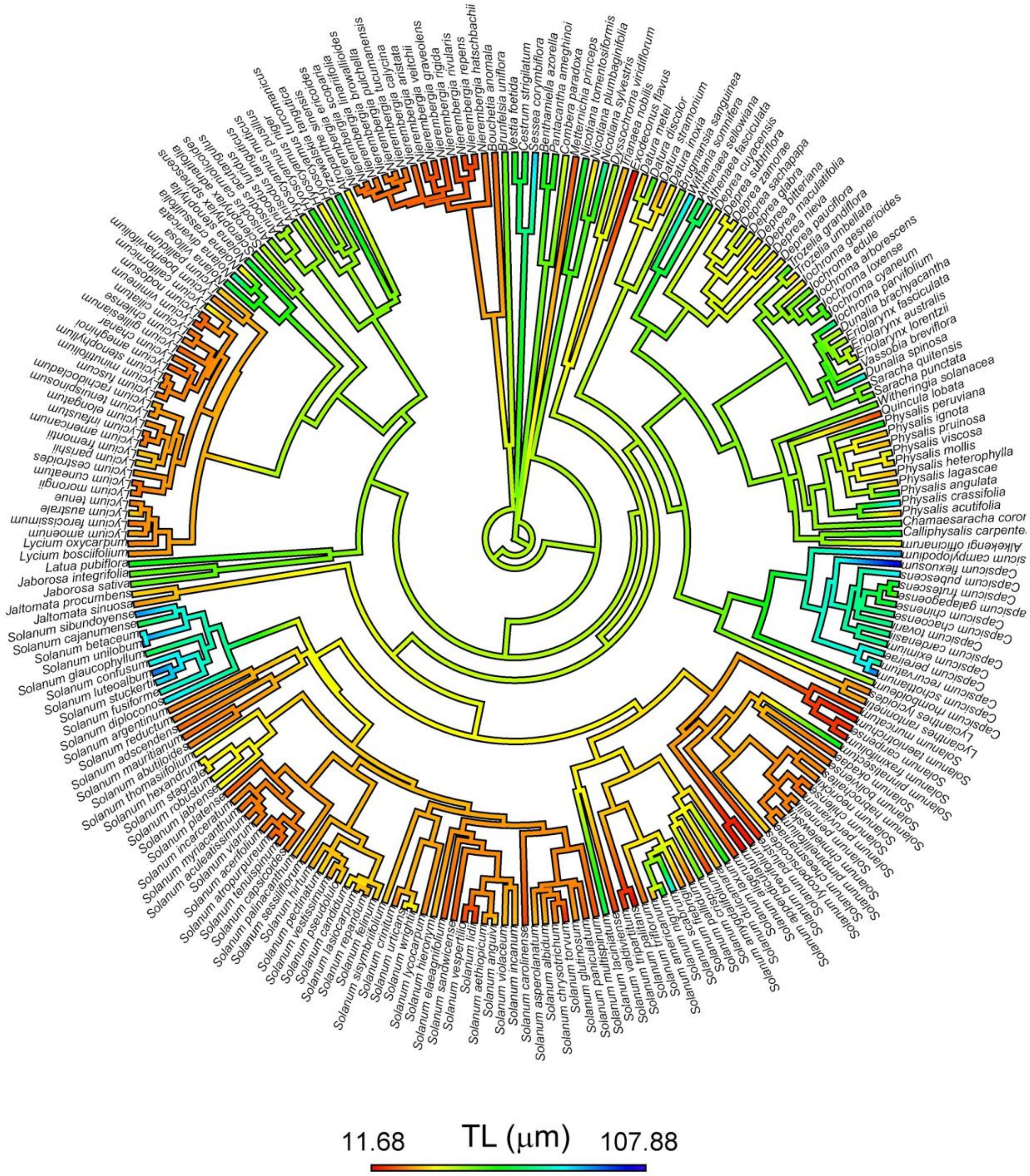

Supplement: Supplementary file 2 [file Image_2.pdf]
