# Supplementary material for: Chromosome Evolution in the Family Solanaceae
Source: Front Plant Sci. 2022 Jan 28;12:787590. doi: 10.3389/fpls.2021.787590 (PMC8832121; doi:10.3389/fpls.2021.787590)

Figure S3. Heatmap of Mean Arm Ratio (r) reconstructed on Solanaceae. Scale below indicates values of r and its color guide.

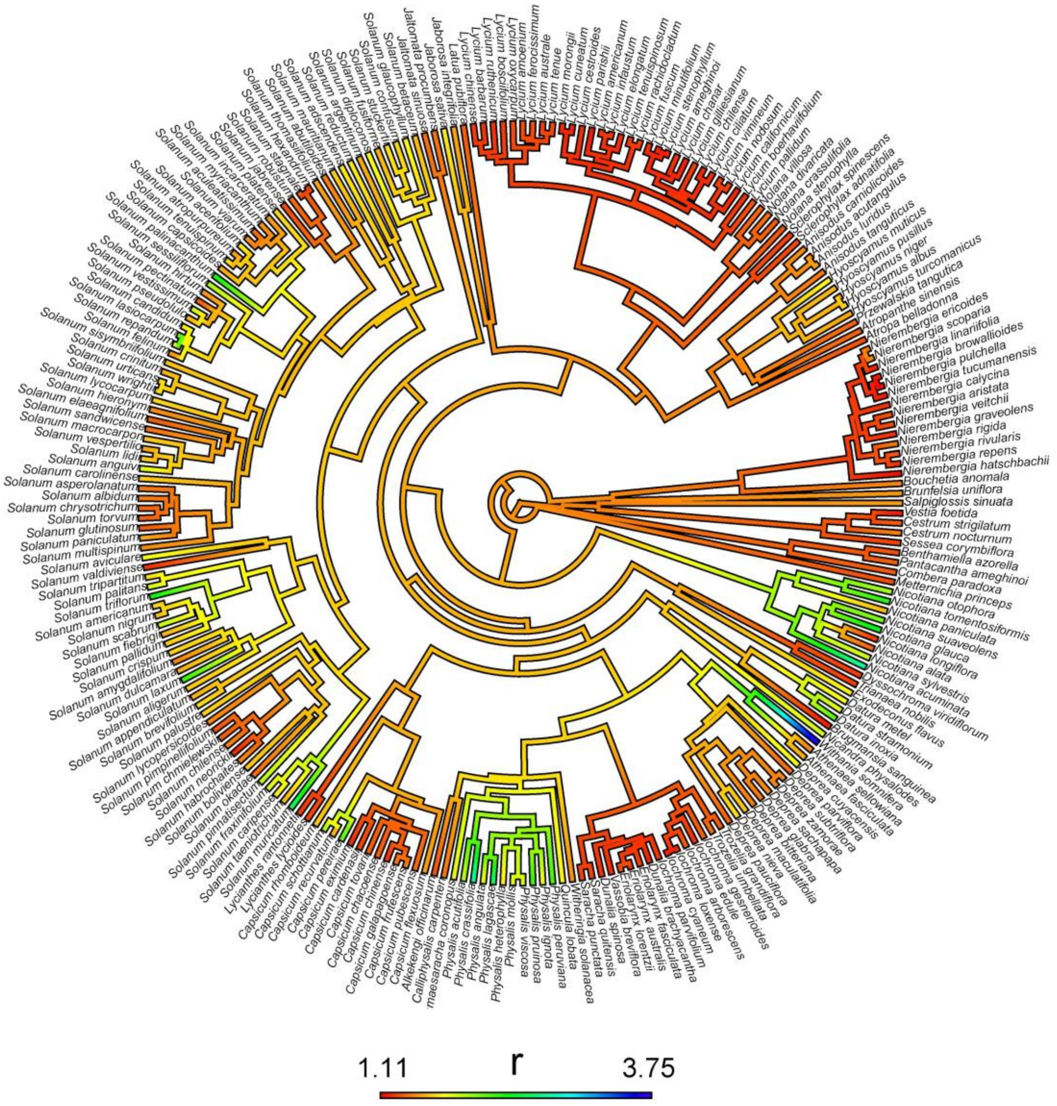

Supplement: Supplementary file 3 [file Image_3.pdf]
