# Supplementary material for: Chromosome Evolution in the Family Solanaceae
Source: Front Plant Sci. 2022 Jan 28;12:787590. doi: 10.3389/fpls.2021.787590 (PMC8832121; doi:10.3389/fpls.2021.787590)
Supplement: Supplementary file 11 [file Table_7.pdf]

SUPPLEMENTARY MATERIAL

Table S7. Summary of the main chromosomal data available for Solanaceae with karyotypes. Proportions calculated over the total species number according to our data revision by 1st November 2020. Highlighted in bold the nine genera with the highest number of species

Subfamily	Tribe	Subtribe/ Clade	Genus (# spp)	counts (%)	karyotypes (%)	spp with band (%)	spp with FISH (%)
Benthamielleae			*Benthamiella* (11)	3 (27.27)	3 (27.27)	0 (0)	2 (18.18)
Cestroideae	Cestreae		***Cestrum* (233)**	**27 (11.59)**	**20 (8.58)**	**8 (3.43)**	**19 (8.15)**
			Sessea (22)	3 (13.64)	2 (9.09)	0 (0)	1 (4.54)
			Vestia (1)	1 (100)	1 (100)	0 (0)	1 (100)
Nicotianoideae			***Nicotiana* (73)**	**60 (82.19)**	**4 (5.48)**	**8 (10.96)**	**31 (42.47)**
Petunioideae	Petunieae		*Fabiana* (16)	5 (31.25)	2 (12.5)	0 (0)	1 (6.25)
			Bouchetia (3)	2 (66.67)	1 (33.33)	1 (33.33)	1 (33.33)
			Leptoglossis (7)	2 (28.57)	1 (14.29)	1 (16.67)	1 (14.29)
			Nierembergia (22)	17 (77.27)	17 (77.27)	15 (71.43)	17 (77.27)
			Petunia (19)	13 (68.42)	2 (10.53)	2 (10.53)	2 (10.53)
Solanoideae	Atropina	Lycieae	***Lycium* (105)**	**48 (45.71)**	**46 (43.81)**	**24 (22.86)**	**35 (33.33)**
			Jaborosa (22)	16 (72.73)	16 (72.73)	15 (65.22)	15 (65.22)
			Sclerophylax (13)	4 (30.77)	4 (30.77)	4 (28.57)	4 (30.77)
	Capsiceae		*Capsicum* (41)	30 (73.17)	25 (60.98)	25 (60.98)	15 (36.59)
			***Lycianthes* (161)**	**20 (12.42)**	**5 (3.11)**	**1 (0.62)**	**2 (1.249)**
	Hyoscyameae		*Atropa* (5)	3 (60)	1 (20)	0 (0)	1 (20)

			Hyoscyamus (15)	10 (66.67)	8 (53.33)	0 (0)	1 (25)
	Physalideae	Iochrominae	*Dunalia* (4)	3 (75)	3 (75)	0 (0)	3 (75)
			Eriolarynx (4)	3 (75)	3 (75)	3 (100)	3 (75)
			Iochroma (35)	8 (22.86)	8 (22.86)	7 (22.58)	8 (22.86)
			Saracha (4)	2 (50)	2 (50)	2 (66.67)	2 (50)
			Trozelia (2)	2 (100)	2 (100)	0 (0)	2 (100)
			Vassobia (2)	1 (50)	1 (50)	1 (50)	1 (50)
		Physalidinae	***Physalis* (109)**	**32 (29.36)**	**27 (24.77)**	**5 (4.59)**	**6 (5.50)**
			Witheringia (12)	2 (16.67)	2 (16.67)	0 (0)	1 (8.33)
		Withaninae	***Deprea* (53)**	**15 (28.3)**	**15 (28.3)**	**10 (18.87)**	**3 (5.66)**
			Tubocapsicum (1)	1 (100)	1 (100)	2 (100)	1 (100)
			Withania (10)	5 (50)	2 (20)	2 (20)	2 (20)
	Solandreae		*Dyssochroma* (3)	2 (66.67)	2 (66.67)	1 (33.33)	0 (0)
			Trianaea (3)	1 (33.33)	1 (33.33)	0 (0)	1 (33.33)
	Solaneae		***Jaltomata* (73)**	**8 (10.96)**	**2 (2.74)**	**1 (1.41)**	**1 (1.37)**
			***Solanum* (1238)**	**506 (40.87)**	**278 (22.46)**	**69 (5.57)**	**87 (7.02)**
			Exodeconus (6)	4 (66.67)	4 (66.67)	4 (66.67)	4 (66.67)
			***Nolana* (92)**	**8 (8.7)**	**5 (5.43)**	**3 (3.26)**	**4 (4.35)**
