# Supplementary material for: Chromosome Evolution in the Family Solanaceae
Source: Front Plant Sci. 2022 Jan 28;12:787590. doi: 10.3389/fpls.2021.787590 (PMC8832121; doi:10.3389/fpls.2021.787590)
Supplement: Supplementary file 12 [file Table_8.pdf]

Table S8. Genera of Solanaceae without chromosomal information of any kind. Highlighted in red the five largest ones.

Subfamily	Tribe	Clade	Genus (number of spp)
Cestroideae	Salpiglossideae		*Reyesia* (4)
	Browallieae		*Protoschwenkia* (1)
Duckeodendroideae			*Duckeodendron* (1)
Goetzoideae			*Goetzea* (2)
			Coeloneurum (1)
			Henoonia (1)
			Tsoala (1)
Petunioideae			*Plowmania* (1)
Schwenckioideae			*Melananthus* (5)
			Heteranthia (1)
Solanoideae	Datureae		*Trompettia*
	Physalideae	Indeterminate	*Darcyanthus* (1)
		Indeterminated	***Cuatresia* (16)**
		Physalidinae	***Physaliastrum* (9)**
			Tzeltalia (3)
			Brachistus (2)
			Capsicophysalis (1)
			Oryctes (1)
		Withaninae	*Nothocestrum* (4)
			Discopodium (1)
			Mellissia (1)
	Solandreae		***Markea* (21)**
			***Juanulloa* (10)**
			***Schultesianthus* (8)**
			Hawkesiophyton (4)
			Merinthopodium (3)
			Poortmannia (1)
