# Supplementary material for: Chromosome Evolution in the Family Solanaceae
Source: Front Plant Sci. 2022 Jan 28;12:787590. doi: 10.3389/fpls.2021.787590 (PMC8832121; doi:10.3389/fpls.2021.787590)

Figure S4. Heatmap of m ratio (proportion of metacentric chromosomes over the total chromosomes of each karyotype formula) reconstructed on Solanaceae. Scale below indicates values of the ratio and its color guide. Values vary from 0 (no metacentric chromosomes) to 1 (all chromosomes metacentric).

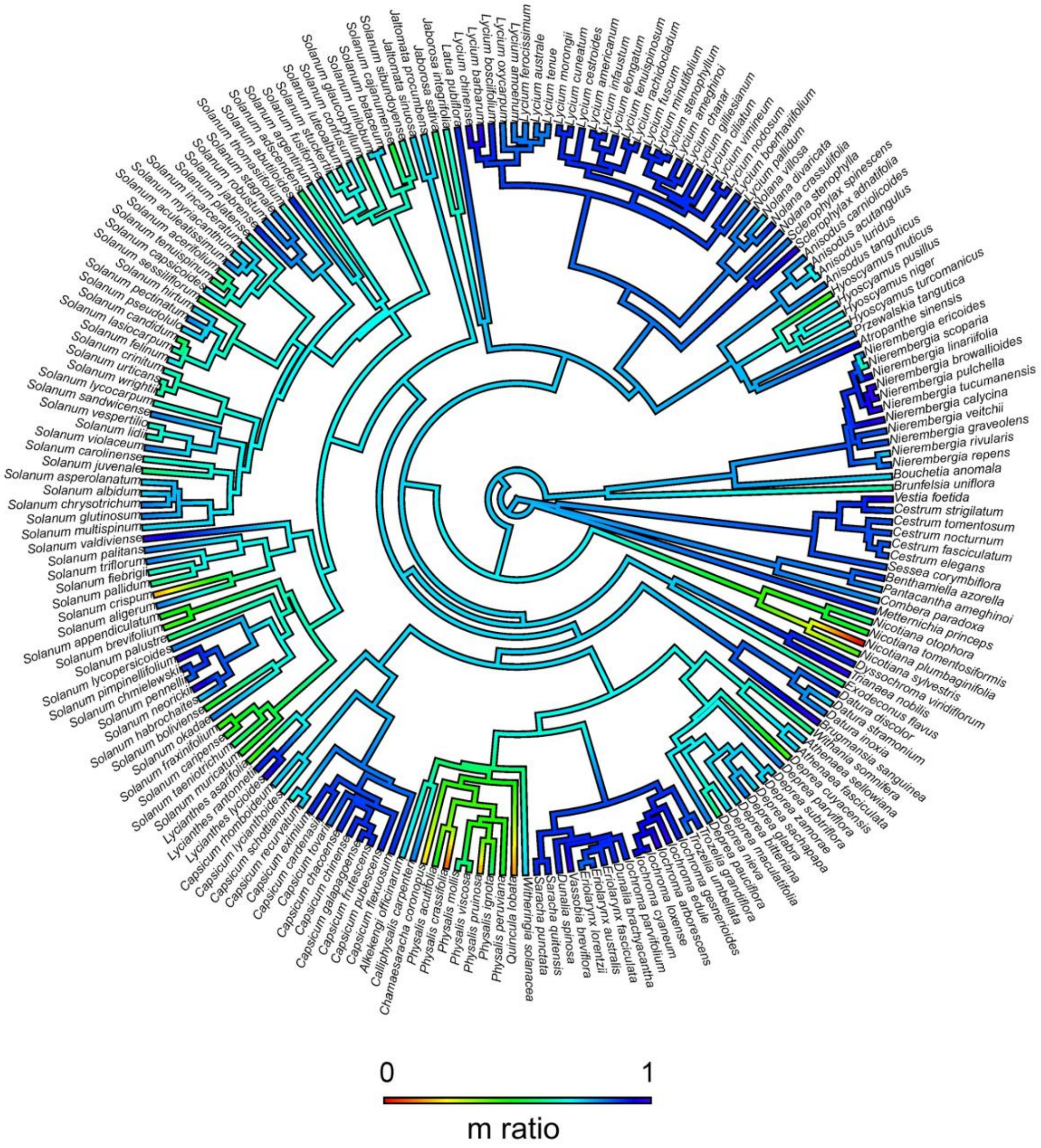

Supplement: Supplementary file 4 [file Image_4.pdf]
